# *Staphylococcus aureus* phagocytosis is affected by senescence

**DOI:** 10.3389/fragi.2023.1198241

**Published:** 2023-07-31

**Authors:** Esteban Robledo, Paula Guadalupe Benito Rodriguez, Israel Aníbal Vega, María Isabel Colombo, Milton Osmar Aguilera

**Affiliations:** ^1^ Instituto de Histología y Embriología (IHEM) “Dr. Mario H. Burgos” CONICET, Universidad Nacional de Cuyo Mendoza, Mendoza, Argentina; ^2^ Departamento Bases Científicas en Salud-Facultad de Ciencias Médicas, Facultad de Medicina, Biología Celular y Molecular, Universidad Nacional de Cuyo, Mendoza, Argentina; ^3^ Departamento de Biología, Facultad de Ciencias Exactas y Naturales, Universidad Nacional de Cuyo, Mendoza, Argentina; ^4^ Facultad de Odontología, Microbiología, Parasitología e Inmunología, Universidad Nacional de Cuyo, Mendoza, Argentina

**Keywords:** aging, senescence, endophagocytic pathway, *Staphylococcus aureus*, phagocytosis

## Abstract

Senescent cells accumulate in multicellular animals with aging, resulting in organ or tissue dysfunction. These alterations increase the incidence of a variety of illnesses, including infectious diseases, and, in certain instances, its severity. In search of a rationale for this phenomenon, we focused on the endophagocytic pathway in senescent cells. We first described the endocytic vesicle populations at different stages of maturation using confocal microscopy. There was an increase in the number of vacuoles per cell, which was partially explained by an increase in cell size. No changes in vesicle maturation or degradation capacities were determined by microscopy or Western blot assays. Also, we studied the internalization of various endophagocytic cargoes in senescent cells and observed only a decrease in the intracellular recovery of bacteria such as *Staphylococcus aureus*. Afterwards, we studied the intracellular traffic of *S. aureus*, and observed no differences in the infection between control and senescent cells. In addition we quantified the recovery of bacteria from control and senescent cells infected in the presence of several inhibitors of endophagosomal maturation, and no changes were observed. These results suggest that bacterial internalization is affected in senescent cells. Indeed, we confirmed this hypothesis by determining minor bacterial adherence and internalization by confocal microscopy. Furthermore, it is important to highlight that we found very similar results with cells from aged animals, specifically BMDMs. This alteration in senescent cells enlightens the diminished bacterial clearance and may be a factor that increases the propensity to suffer severe infectious conditions in the elderly.

## 1 Introduction

Multiple defense mechanisms exist in multicellular organisms to prevent the spread of single-cell DNA damage. Among them, cell death by apoptosis or necrosis, blockage of cell replication by activating senescence, and remodeling of the cytoplasm by autophagy may take place. Phagocytosis is the process that eventually eliminates senescent, apoptotic, and necrotic cells. The initial reports about senescence date back to 1961, when Hayflick and Moorehead described alterations of *in vitro* cultured cells after several passages (Hayflick and Moorhead, 1961)⁠. DNA damage is the primary stimulus that induces senescence, but oxidative stress, mitochondrial damage, hypoxia, and oncogene activation could also activate this pathway (Chen et al., 1995; Muñoz-Espín and Serrano, 2014; Roy et al., 2020)⁠. Activating senescence results in morphological and functional alterations in cells (Campisi and D’Adda Di Fagagna, 2007)⁠. The list of cell changes includes resistance to apoptotic signals, arrest of which proliferation, and changes in gene expression (Campisi and D’Adda Di Fagagna, 2007)⁠.

Senescent activation is not defined by a particular characteristic. Nevertheless, the accumulation of senescence-associated β-galactosidase (SA-β-gal) and the increase in lysosomal SA-β-gal activity are senescence markers widely used (Dimri et al., 1995; Lee et al., 2006; Debacq-Chainiaux et al., 2009)⁠. *In vitro*, the senescent phenotype is characterized by altered nuclear morphology and chromatin structure as well as increased cell size (Narita et al., 2003; Rai and Adams, 2012)⁠. Another characteristic of senescence activation is a process called senescence-associated secretory phenotype (SASP) (Coppé et al., 2008; Byun et al., 2015)⁠, where cells secrete several factors affecting tissue remodeling, inflammation, and cell growth (Kuilman and Peeper, 2009; Freund et al., 2010; Basisty et al., 2020)⁠. One of the functions of SASP is to modulate the immune system in order to promote the elimination of senescent cells (Weisheit et al., 2015; Casiraghi et al., 2016)⁠. But if the signal persists, non-damaged cells may undergo senescence (Acosta et al., 2013)⁠.

The deterioration of the immune system with aging causes a disfunction in immunological surveillance (Schneider, 1983)⁠ increasing the prevalence and severity of infectious diseases (Aw et al., 2007; Pawelec, 2012; Martín et al., 2017)⁠. This phenomenon, known as immunosenescence, may increase the risk of *Staphylococcus aureus* infections among the elderly (McClelland et al., 1999; Kang et al., 2011; Thorlacius-Ussing et al., 2019)⁠. In addition, the clinical manifestations are more severe in older adults compared to younger individuals (McClelland et al., 1999; Tacconelli et al., 2006; Bassetti et al., 2018)⁠.

The endophagocytic pathway refers to the process by which cells ingest extracellular molecules or particles and degrade them within lysosomes (Huotari and Helenius, 2011)⁠. After the material has been taken in by the cell, a vesicle called an “endosome” is formed. The endosome fuses with lysosomal vesicles containing digestive enzymes that degrade the vesicle’s contents (Huotari and Helenius, 2011; Scott et al., 2014)⁠. Phagocytosis, pinocytosis, and clathrin-mediated endocytosis are three distinct types of endocytosis, each of which involves the ingestion of foreign components through slightly different molecular mechanisms (Huotari and Helenius, 2011)⁠. Endocytosis is essential for a wide range of physiological processes, including nutrient uptake, waste elimination, and the internalization of signaling molecules. Phagocytosis is characterized by the uptake of large extracellular particles, such as microorganisms or dead cells (Gordon, 2016; Lancaster et al., 2019; Uribe-Querol and Rosales, 2020)⁠, and this mechanism may change when immunosenescence occurs. Macrophages from older patients have a reduced phagocytic function (De La Fuente, 1985; Khare et al., 1996; De La Fuente et al., 2000)⁠, Interestingly, a similar change has been observed in aged *Drosophila melanogaster* hemocytes (Horn et al., 2014)⁠.

Autophagy is an important pathway connected to the endolysosomal pathway that maintains cellular homeostasis by eliminating damaged proteins and organelles (Mizushima et al., 2008)⁠. This process begins when a double membrane structure closes and sequesters cytoplasmic material such as protein aggregates, ribosomes, and even whole organelles like mitochondria (Eskelinen, 2005)⁠. These vesicles fuse sequentially with late endosomes and lysosomes, forming an autolysosome where the sequestered material is degraded (Eskelinen, 2005)⁠.

Numerous microorganisms are capable of surviving and replicating within phagocytic cells. They have developed a variety of protection strategies, like preventing the maturation of phagosomes or damaging this compartment letting the bacteria escape into the cytoplasm (Flannagan et al., 2009)⁠. In infections caused by *S. aureus,* a large number of bacteria are degraded following phagocytosis and those that survive. After that, the bacteria lysate the vacuole and then the cell, causing the bacterial spreading (Kubica et al., 2008)⁠.

Multiple studies have demonstrated that the autophagic pathway is essential for the survival and multiplication of *S. aureus* within cells. These studies demonstrated that the vacuoles harboring the bacteria are modified autophagosomes that do not become degradative compartments (Schnaith et al., 2007; Mestre and Colombo, 2012)⁠.

In the current report, we focused on the endophagocytic pathway in senescent epithelial and phagocytic cells. Surprisingly, after examining vesicle populations with confocal microscopy, we observed an increase in vacuole counts in all cases when senescence is activated. However, there were no alterations in vesicle maturation or degradation capacity. Next, we examined the internalization of various endophagocytic cargoes, observing a decrease in the intracellular recovery of the bacteria *S. aureus*. Then, we noticed that a decrease in bacterial internalization rather than an increase in bacterial degradation inside the cell was the cause of the observed effect. The alteration of *S. aureus* phagocytosis was also observed in Raw monocytes, which are professional phagocytes. Finally, using primary cultures of macrophages from aged animals, we found the same phagocytosis defects, confirming our hypothesis. Our results suggest that due to senescence activation-related defects in the early phagocytic process, bacterial clearance could be diminished in an organism. This failure may contribute to the observed susceptibility of the elderly to severe infectious diseases such as sepsis.

## 2 Methods

### 2.1 Reagents and antibodies

For specific assays, cells were treated with Bafilomycin A1 (Merck Milllipore, # 11707), NH4Cl (Merck Milllipore, # A9434-500G), and tert-butyl hydroperoxide (Merck Milllipore, # 458139). For specific transfections, we used EGFP-tagged human Rab5, which was kindly provided by Philip D. Stahl (Washington University, St. Louis), and EGFP-RAB7A, which was given by Bo van Deurs (University of Copenhagen, Denmark). In the current work, we used the following antibodies: rabbit antiserum against MAP1LC3B (Sigma-Aldrich # L7543, RRID:AB_796155), rabbit anti-cathepsinD (Dako, #A0561), mouse anti-LAMP2 (Santa Cruz Biotechnology, # sc-18822, RRID:AB_626858), mouse anti-tubulin (Abcam # ab7750, RRID:AB_306044), and mouse monoclonal anti-actin (Sigma-Aldrich, # A5441, RRID:AB_476744). The secondary antibodies used in the work were: HRP-conjugated goat anti-rabbit antiserum (Jackson ImmunoResearch Labs, # 111-035-003, RRID:AB_2313567), HRP-conjugated goat anti-mouse antiserum (Jackson ImmunoResearch Labs, # 115-035-003, RRID: AB_10015289), and Alexa Fluor 573-conjugated goat anti-mouse antiserum (Molecular Probes, # A-21424, RRID:AB_141780).

### 2.2 Cell culture, drug treatments and transfection

HeLa cells were cultured in Gibco Dulbecco’s Modified Eagle Medium (ThermoFisher Scientific # 12800-058), while CHO GFP-LC3 and Raw cells were grown in alpha MEM (ThermoFisher Scientific # 10800-058) medium supplemented with 10% heat-inactivated fetal bovine serum (FBS, Natocor), 2.2 g/L sodium bicarbonate, 2 mM glutamine, and 0.1% penicillin-streptomycin (ThermoFisher Scientific # 15140148). Cells were maintained at 37°C in a 5% CO_2_ atmosphere. Cells were subcultured after trypsinization (0.025% trypsine-EDTA, ThermoFisher Scientific, #15400054). For senescence activation, HeLa cells were grown to reach 80% confluence and then treated with tert-butyl hydroperoxide at 250 μM for 2 h. Subsequently, cells were washed and incubated in D-MEM + 10% FBS for 5 days. To perform autophagy experiments, control or infected cells were incubated at 37°C for 2 h in the presence or absence of 100 nM bafilomycin A1. Afterwards, cells were lysed and processed to be analyzed by Western blot. For transient expressions, Lipofectamine 3000 (ThermoFisher Scientific, # L3000008) was used according to the manufacturer’s instructions.

### 2.3 Cell primary culture

Vascular smooth muscle cells (VSMC) derived from mesenteric arteries were isolated using a method previously described (Touyz et al., 1994; Gil Lorenzo et al., 2014)⁠. Cells were obtained from Wistar-Kyotto (WKY) male rats (8–10 weeks old). VSMCs were identified by positive antibody staining against α-actin between the second and ninth passages. The procedures were carried out on subconfluent (50%–90%) cultures.

Following a previously described protocol (Weischenfeldt and Porse, 2008; Zhang et al., 2008; Vanrell et al., 2022)⁠, bone marrow cells were obtained from the femur bones of C57BL/6J WT mice and suspended in cold alpha MEM containing 40 g/mL of gentamicin. These bone marrow progenitor cells were isolated in 100-mm plates containing 10% FBS, 40 g/mL gentamicin, 2 mM L-glutamine, and conditioned medium derived from a 4-day culture of 30% L929 cells. The cells were then washed, and the same medium was added for an additional 6 days, leading to macrophage differentiation. Finally, macrophages were identified using CD11B and F480 as markers.

### 2.4 Cell staining, visualization, and image processing

For immunofluorescence, HeLa cells were grown on coverslips overnight to reach 50%–80% confluence. After incubation under different experimental conditions, cells were fixed with 3% paraformaldehyde (Merck Millipore, 158127) in PBS for 10 min at 37°C, washed with PBS, and reacted groups blocked with 50 mM NH4Cl (Merck Millipore, #213330) in PBS. Subsequently, they were permeabilized with 0.05% saponin (Sigma-Aldrich, #S4521) in PBS containing 0.5% BSA (Sigma-Aldrich, # A2153) and mounted in MOWIOL (Merck Milllipore, # 475904-M). Fixed cells were analyzed in an Olympus Fluoview 1000 confocal microscope using a 60x PlanApo oil NA 1.42 objective. All images were processed with the Fiji ImageJ software (Wayne Rasband, National Institutes of Health, RRID:SCR_002285). For the colocalization analysis, a single plane image from each channel was acquired, processed as described before, and then the Manders coefficient was calculated with Fiji ImageJ.

### 2.5 SA-β-galactosidase staining

HeLa cells were stained for SA-β-glactosidase according to the manufacturer’s instructions (Cell Signaling Technologies, catalog number 9860). Briefly, cells were washed three times with PBS and then fixed with 3% paraformaldehyde (Merck Millipore, 158127) in PBS for 15 min at room temperature. After two PBS washes, a β-galactosidase staining solution containing 1 mg/mL X-gal at a pH of 6.0 was added to the cells, and the culture plate was sealed with parafilm and kept at 37°C in a dry incubator overnight. The β-galactosidase staining solution was washed away and substituted by Mowiol. Pictures were captured at ×20 magnification using a Nikon DS-Ri2 camera and a Nikon Ti wide-field light microscope from Tokyo, Japan.

### 2.6 Western blots

Cells were lysed for 30 min at 4°C with RIPA buffer supplemented with the following protease activity inhibitors: 10 μg/mL aprotinin, 10 μg/mL leupeptin, 5 μg/mL pepstatin A, 1 mM sodium orthovanadate, and 1 mM sodium fluoride. Lysates were treated with Laemmli’s buffer and separated by SDS-PAGE. After electrophoresis, proteins were transferred onto nitrocellulose at 200 mA for 1 h at room temperature. Protein bands were detected by incubating membranes with the specific primary antibody for 1 h at room temperature, followed by a washing step with PBS containing 0.05% Tween 20, and then incubation with an HRP-conjugated secondary antibody for 1 h. Membranes were washed with PBS-Tween20 and then incubated with the Enhanced Chemiluminescence (ECL) reagent (General Electric, # RPN2232) and analyzed with Fujifilm LAS-4000 equipment. The densitometric analysis of the bands was performed with the ImageJ software.

### 2.7 Statistical analysis

Results are presented as the mean ± SEM from three independent experiments. T-test or ANOVA in conjunction with Tuckey and Dunnett tests were used to perform sample comparisons. Significant differences: *p < 0.01; **p < 0.005; ***p < 0.001.

## 3 Results

### 3.1 Senescence increases the number of endocytic vesicles

**FIGURE 1:**
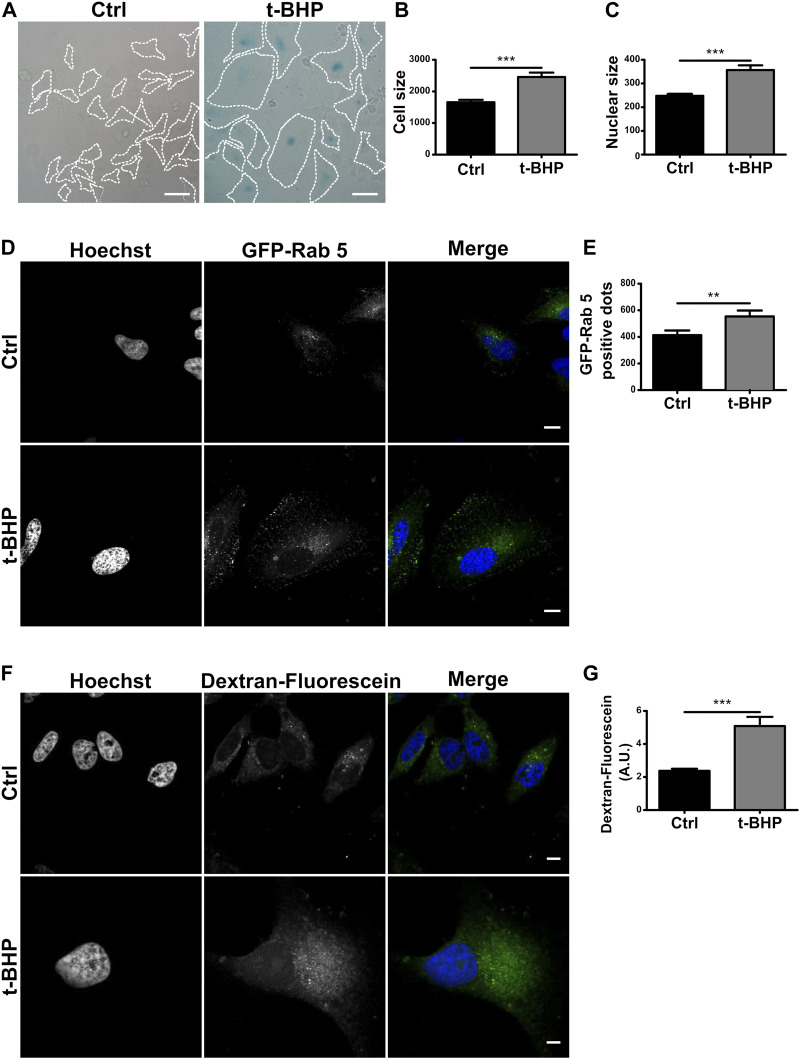
Senescence increases the number of endosomes per cell. **(A)** HeLa cells were treated with t-BHP for 2 h, then washed and cultured for 5 days to induce senescence. Next, both control and senescent cells were fixed and stained with X-gal (Scale bar, 50 um). **(B)** Measurement of cellular size. [*n* = 3 (in each experiment 60 cells were examined), mean ± SEM ***p < 0.0001] **(C)** Hoechst was used to stain control and senescent cells to visualize DNA. Nuclear size in untreated and t-BHP-treated cells was measured [*n* = 3 (60 cells were analyzed in each triplicate), mean ± SEM ***p < 0.0001]. **(D)** Cells treated and untreated with t-BHP were transfected with GFP-Rab5 and analyzed by confocal microscopy 24 h after transfection. **(E)** The number of Rab5 positive dots was quantified [*n* = 3 (35 cells in each triplicate), mean ± SEM *p = 0.0148.] (Scale bar, 10 μm). t-BHP-treated and untreated cells were incubated with dextran-fluorescein for 2 h, then fixed and analyzed by confocal microscopy. Subsequently, the dextran-fluorescein fluorescence intensity per cell was quantified [*n* = 3 (32 cells were analyzed for each experimental triplicate), mean ± SEM ***p < 0.0001] (Scale bar, 10 μm).

In the context of senescence activation, the endocytic pathway has received little attention. Previous studies have mainly concentrated on endocytic changes that lead to senescence (Shin et al., 2021). In this study, we were interested in investigating whether senescence impacts the endophagosomal pathway. Senescence is divided into replicative and stress-induced premature senescence (SIPS) categories (Toussaint et al., 2000)⁠. The latter refers to the activation of senescence by a stimulus like UV radiation or oxidative stress. *In vitro*, H2O2 or analogs such as tert-butylhydroperoxide (tBHP) treatment generally induce SIPS (Toussaint et al., 1992; Duan et al., 2005)⁠. Thus, we used tBHP to induce senescence in HeLa cells. The activation of the pathway was then assessed by staining the senescence-associated β-galactosidase which is known to mark predominantly senescent cells but not quiescent cells (Figure 1A). In order to corroborate the activation, we also evaluated some phenotypical characters such as cell and nucleus dimensions (Figures 1B, C), observing a marked size increase in both cells and nuclei, in agreement with previous publications (Gottwald et al., 2020)⁠.

To examine endosome populations in senescent cells, we initially use the early endosome marker Rab5, a small GTPase that binds to the membrane of early endosomes and is one of the most frequently used endocytic markers (Numrich and Ungermann, 2014; Wandinger-Ness and Zerial, 2014)⁠ of HeLa cells overexpressing GFP-Rab5 (Figure 1D), the number vacuoles increased in senescent cells compared to control cells (Figure 1E). In addition, we investigated the cells ability to internalize dextran particles marked with fluorescein. This particle is not degraded, so it accumulates in endosomes at different stages of maturation, allowing for monitoring the entire pathway, including endolysosomes. Similar to the above results, senescence increased the number of vesicles containing fluorescent dextran (Figures 1F, G).

### 3.2 Endosome maturation is not affected in senescent cells

**FIGURE 2:**
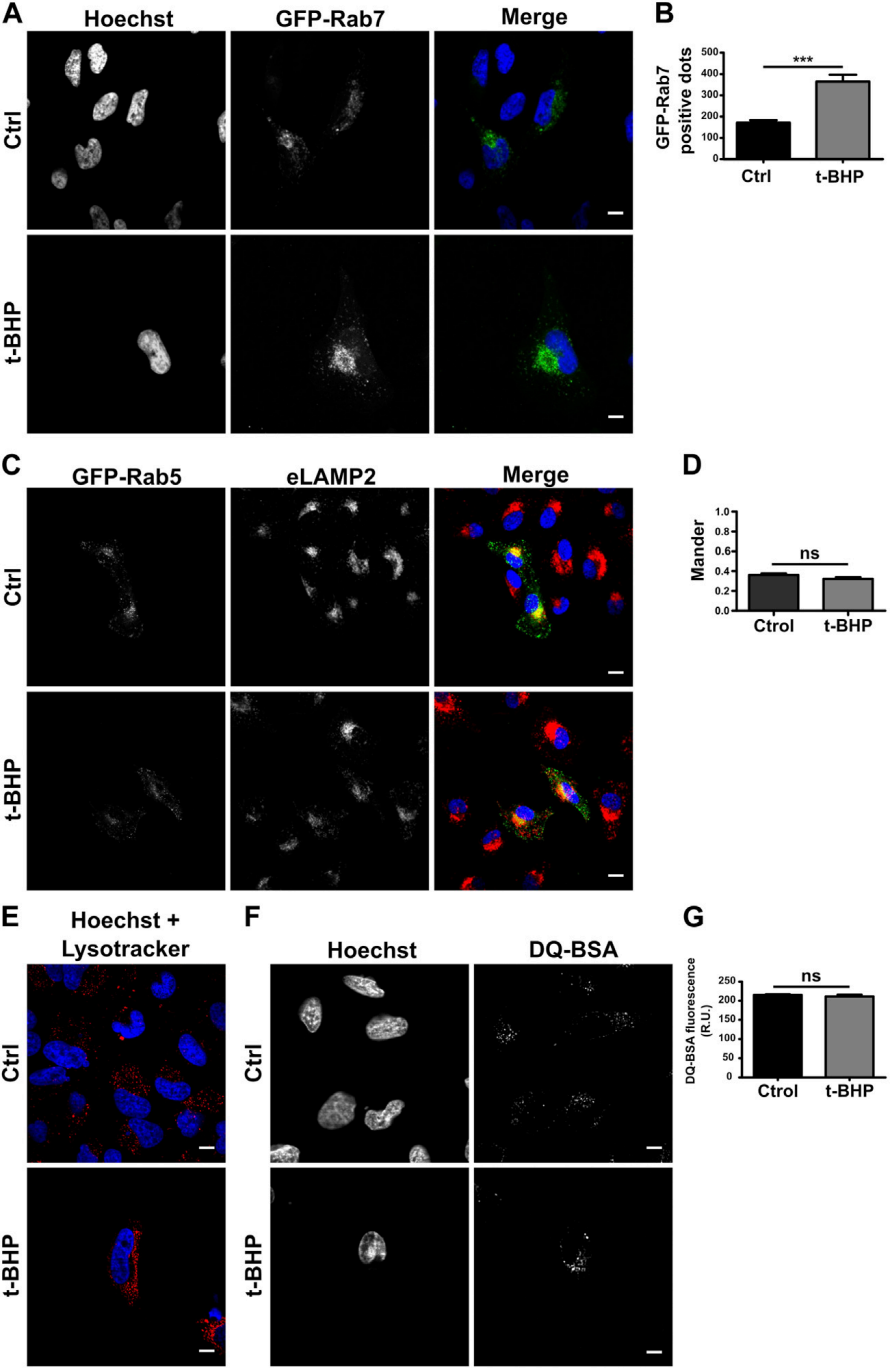
Senescence has no effect on endosome maturation. **(A)** HeLa cells treated or untreated with t-BHP were transfected with GFP-Rab7 and examined 24 h after transfection using confocal microscopy. **(B)** The number of Rab7-positive dots was quantified [*n* = 3 (each n tested = 33 cells), mean SEM ***p 0.0001]. **(C)** Both t-BHP-treated or untreated cells were transfected with GFP-Rab5, and 24 h after transfection, cells were fixed and immunostained with an anti-LAMP2 antibody and a specific secondary antibody. **(D)** Samples were analyzed by confocal microscopy, and colocalization of GFP-Rab5 with LAMP2 was calculated [*n* = 3 (each n tested = 45 cells), mean ± SEM ns *p* = 0.1938]. **(E)** Control and senescent cells were stained with LysoTracker red and then analyzed by confocal microscopy. **(F)** Evaluation of control and senescent cells incubated with DQ-BSA. **(G)** Quantification of DQ BSA fluorescence intensity from confocal microscopy images [*n* = 3 (each n tested 42 cells), mean ± SEM ns *p* = 0.4049] (Scale bar, 10 μm).

When the maturation of the endocytic pathway is blocked, early endosomes accumulate. To determine whether the increased quantity of vesicles is due to an endosome maturation defect, we monitored mature endosomes overexpressing GFP-Rab7 (Figure 2A) or detecting endogenous LAMP2 (Supplementary Figure S1A), two markers of late endosomes (Numrich and Ungermann, 2014; Wandinger-Ness and Zerial, 2014)⁠. When the number of labeled vesicles was determined, senescent cells also contained a greater number of Rab7 (Figure 2B) or LAMP2 (Supplementary Figure S1B) vesicles than normal cells, suggesting that the transition from early to late endosomes does not seem to be affected. Thus, we hypothesized that changes in cell size might explain the observed increase. Comparing the ratio obtained by dividing the number of LAMP2-positive (Supplementary Figure S1C) or Rab7-positive vesicles (Supplementary Figure S1D) per cell by the size of the cell, no significant difference between normal and senescent cells was observed. These findings suggest that the increased number of endosomes per cell observed in senescent cells is due to their larger size. In addition, we evaluated the colocalization of GFP-Rab5 and eLAMP2 (Figure 2C) or GFP-Rab7 and LAMP2 (Supplementary Figure S1E), and consistent with the previous observation, we did not observe differences between senescent and normal cells in either case (Figure 2D; Supplementary Figure S1F).

To assess whether the endosomes mature properly in senescent cells, LysoTracker red, a molecule that accumulates in acidic compartments, was used to determine the pH of the vesicles. Similar to normal cells, senescent cells feature many LysoTracker-red staining vesicles, indicating that senescence activation has no influence on the acidification process (Figure 2E). Then, we analyzed its functioning, such as its capacity to degrade bovine serum albumin (BSA). We utilized a BSA derivative that was so densely labeled with the green fluorescent BODIPY^®^ FL dye that the fluorescence emission is self-quenched. When DQ-BSA reaches a degradative compartment, it is cleaved, releasing fluorescent fragments. As shown in Figures 2F, G, the activation of senescence does not prevent the accumulation of this fluorescent probe in cells. In addition, the conversion of the lysosomal enzyme pro-cathepsin D to cathepsin D was analyzed. Cathepsins and other lysosomal proteases are transported by vesicles that originate from the trans-Golgi apparatus and become active upon autoprocessing within late endosomes or lysosomes. Cathepsins need to reach an acidic vesicle in order to suffer this cleavage (Supplementary Figure S2A). We examined pro-cathepsin D cleavage by Western blot (Supplementary Figure S2B) and found no variation in the protein fragments generated by normal or senescent cells (Supplementary Figure S2C).

Taken together, our results indicate that the maturation process and the functionality of the endocytic pathway do not seem to be altered in senescent cells.

### 3.3 Senescence does not modify *S. aureus* intracellular trafficking

**FIGURE 3:**
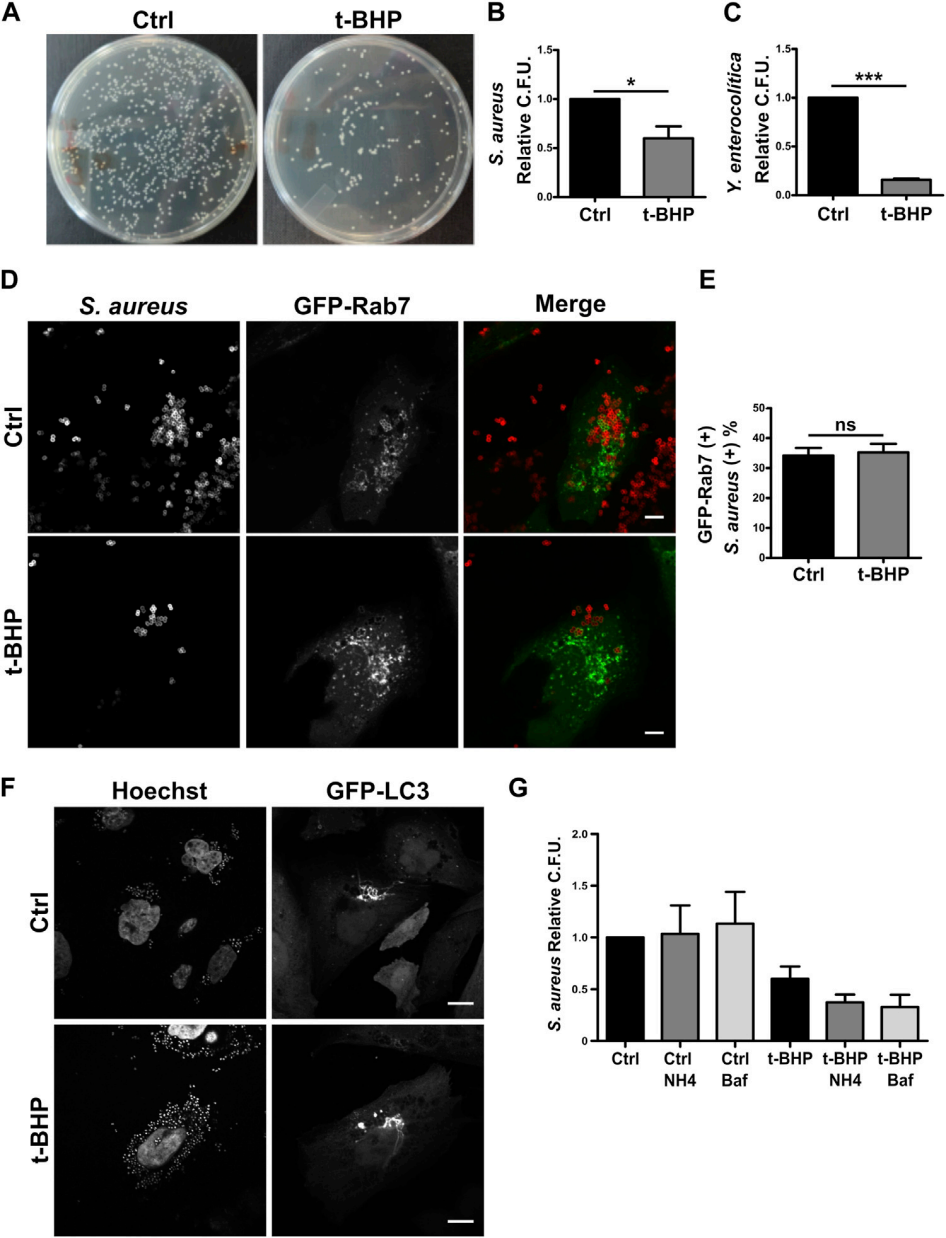
The recovery of bacteria was lower in infected senescent cells than in infected control cells. **(A)** Senescent cells where obtained in accordance with the previously described methodology. Then, both control and senescent cells were infected with *S. aureus* or *Y. enterocolitica* for 1 h. Afterwards, cells were lysed, placed on LB agar, and incubated for 24 h **(B)** *S. aureus* CFU quantification (*n* = 3, mean ± SEM *p = 0.0458) **(C)** *Y. enterocolítica* CFU quantification. (*n* = 3, mean ± SEM *p = 0.0002). **(D)** Confocal microscopy of control and senescent cells, transfected with Rab7, infected with *S. aureus* (M.O.I. = 10), and fixed at 1 h post infection (1hpi) (scale bar = 10 um). **(E)** Quantification of the proportion of bacteria present on GFP-Rab7 positive vesicles in control and senescent cells. [*n* = 3 (each n tested = 30 cells), (Mean ± SEM ns *p* = 0.7878)] **(F)** Confocal microscopy of CHO cells stable transfected with GFP-LC3, treated or untreated with t-BHP, infected with *S. aureus* (M.O.I = 10), and fixed 4 h after infection. (scale bar = 10 μm). **(G)** Control and senescent HeLa cells were pre-treated with inhibitors of endosome maturation, washed, and then infected. Following 10 min of internalization, ClNH4 or BafilomycinA1 was added to the media to block autophagic flux. At 1 hpi, cells were lysed and CFU were measured on LB agar plates. Mean ± SEM. *n* = 3 from three independent experiments.

Defense is an important function of the endo/phagocytic pathway, especially in professional phagocytes like macrophages and neutrophils but also in non-professional phagocytes like epithelial cells (Rabinovitch, 1995; Uribe-Querol and Rosales, 2020)⁠. The function of non-professional phagocytes like epithelial cells *in vivo* is poorly understood. However, epithelial cells have been extensively utilized in the study of bacterial internalization and intracellular events involving a vast array of bacteria. To examine whether senescence influences bacterial phagocytosis in epithelial cells, HeLa cells were infected with gram-positive *S. aureus* or gram-negative *Yersinia enterocolitica* bacteria. After 2 h, cells were lysed, and the recovered bacteria were quantified by counting the colony-forming units (CFU) on the appropriate agar plates (Figure 3A). As indicated in Figure 3B, *S. aureus* recovery was decreased in senescent infected cells. Similarly, the recovery of *Y. enterocolitica* from senescent cells was diminished compared to normal cells (Figure 3C).

Subsequently, due to the bacteria’s clinical relevance, we centered our interest on a more in-depth study of *S. aureus*. Reduced bacterial recovery from senescent cells may be the result of either lower bacterial internalization or higher bacterial degradation within the cell. We first examined *S. aureus* intracellular traffic using confocal fluorescence microscopy. We found no differences between control and senescent cells in the colocalization of bacteria with Rab5 (Supplementary Figures 3A, 3B) or Rab7 (Figures 3D, E).

A hallmark of the intracellular cycle of *S. aureus* is the interaction with the autophagic pathway. Late in the process, the vesicles containing the bacteria fuse to form honeycomb-like structures decorated with the protein LC3 falta nuestra cita. As shown in Figure 3F, senescence does not prevent the formation of such arrangements. The majority of MAP1LC3 is located in the cytosol; however, when autophagy is induced, this protein is cleaved and lipidated, allowing its association with the autophagosome membrane. Due to the differing electrophoretic mobilities of the two forms of MAP1LC3 (MAP1LC3-I, which is non-lipidated, and MAP1LC3-II, which is lipidated), this post-translational processing can be studied by Western blot. It has been shown that following autophagy induction, the level of MAP1LC3B-II increases significantly (Kabeya et al., 2000)⁠. This accumulation is more pronounced when autophagosome maturation (i.e., fusion with lysosomes) is inhibited because the autophagic flux blockage prevents MAPLC3 degradation (e.g., by chloroquine). Similar to the findings shown in previous studies (Luo et al., 2013; Tang et al., 2017; Wang et al., 2018; Kang et al., 2019; Zheng et al., 2019; Chen et al., 2020; Lu et al., 2021)⁠, in HeLa senescent cells we observed a reduction in the autophagic flux under basal conditions (ratio between Baf1 treated and Baf1 untreated conditions, Supplementary Figure S3C, columns 2 and 1). Then, on *S. aureus*-infected cells, the autophagic flux observed in normal and senescent cells did not differ (ratio between Baf1-treated and Baf1-untreated conditions, Supplementary Figure S3C, columns 4 and 3 lines) and seemed to be blocked. These findings suggest that stimulation of senescence does not modify the autophagic pathway related to the infection.

Next, with the purpose of determining whether bacterial degradation within the cell might be altered in senescent cells, we determined the recovered bacteria by counting the colony-forming units (CFU) using various degradation inhibitors. To this end, we infected control and senescent cells in the presence and absence of several phagosome maturation inhibitors. The cells were then lysed, and CFU were used to count the bacteria in relation to the number of cells in each condition and relativized to the number of untreated normal cells (which have the value 1). As shown in Figure 3G, bars 1–3, in the presence of inhibitors, the bacterial recovery of normal cells does not differ significantly from that of untreated cells. Similarly, senescent cells treated with the inhibitors do not exhibit an improvement in bacterial recovery (Figure 3G, bars 4–6). Our findings indicate that senescence does not appear to affect *S. aureus* intracellular trafficking or replication.

### 3.4 Senescent cells fail in the *S. aureus* internalization step

**FIGURE 4:**
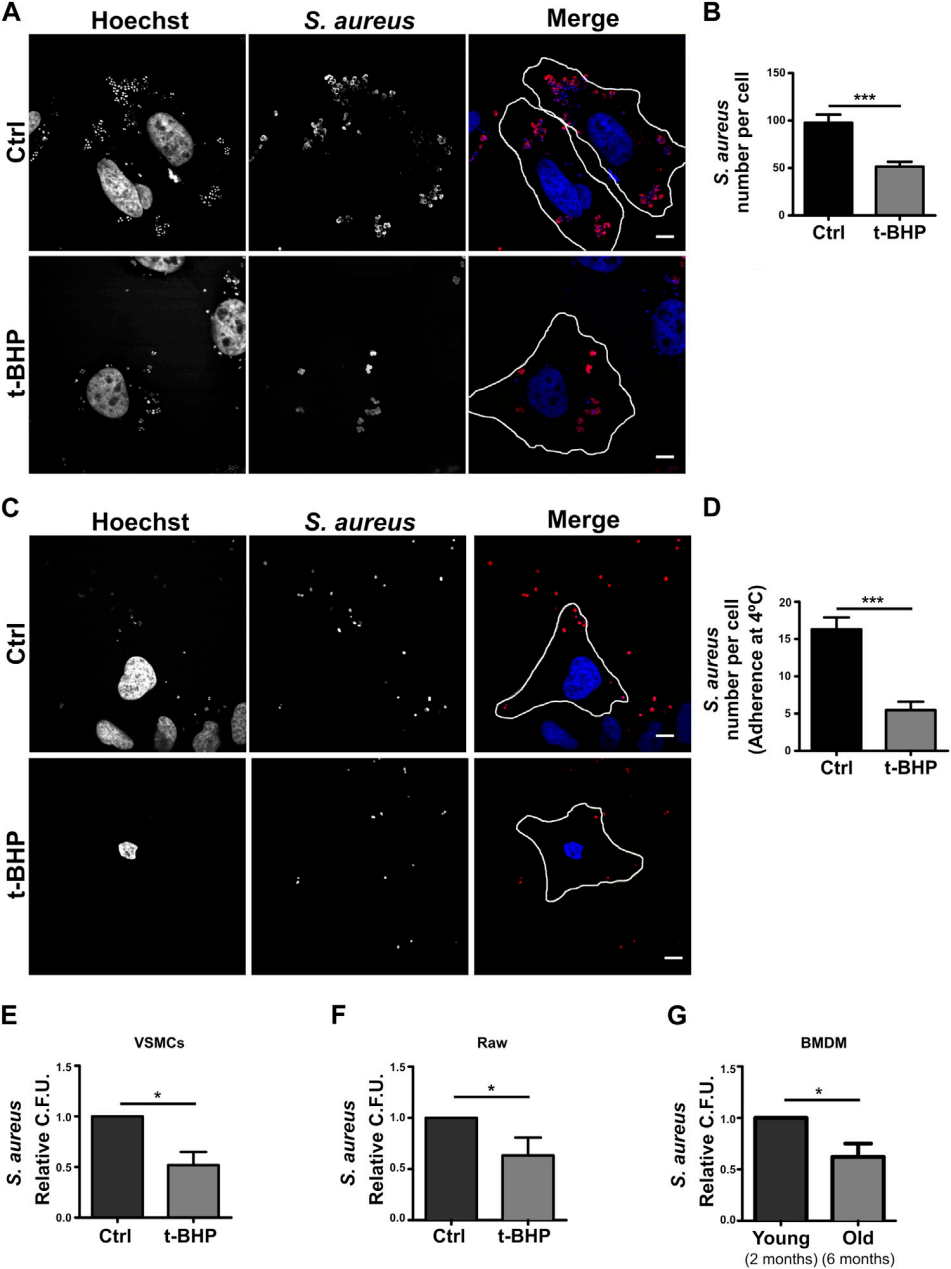
*S. aureus* adhesion and internalization is altered in senescent cells. **(A)** Control and senescent cells were incubated with *S. aureus* for 10 min. Subsequently, extracellular bacteria were removed by a mild trypsin treatment followed by multiple washings. The cells were then fixed 1 h post-infection. M.O.I. = 10 (scale bar = 10 μm). **(B)** Quantification of bacteria per cell. [n = 3 (each n tested = 28 cells) Mean + SEM ***p < 0.0001]. **(C)** Control and senescent cells were incubated with *S. aureus* at 4°C for 30 min and then fixed. M.O.I. = 10 (scale bar = 10 um). **(D)** Quantification of the number of bacteria adhered to the surface of the cell. [*n* = 3 (each n tested 25 cells) Mean + SEM ***p < 0.0001]. **(E)** Senescence induction was performed in VSMCs cell in accordance with the previously described methodology (see Methods). Then, both control and senescent cells were infected with *S. aureus* (MOI = 10) for 1 h. The cells were then lysed, samples placed on LB agar, and incubated for 24 h. *S. aureus* CFU quantification (*n* = 3, mean ± SEM *p = 0.0458). **(F)** Senescence activation was performed in RAW cells as described in methods. Afterwards, both control and senescent cells were infected with *S. aureus* for 1 h. Cells were then lysed, placed on LB agar, and incubated for 24 h. *S. aureus* CFU quantification (*n* = 3, mean ± SEM *p = 0.0458). **(G)** BMDMs from mice of two or 6 months old were infected with *S. aureus* for 1 h. The cells were then lysed, placed on LB agar, and incubated for 24 h. *S. aureus* CFU quantification (*n* = 3, mean ± SEM *p = 0.0458).

Given that our findings revealed that bacterial degradation was not enhanced in senescent cells, we thought that when senescence is induced, the bacterial internalization process may be altered. Using confocal microscopy, this hypothesis was verified with a methodology that eliminates extracellular bacteria with a mild trypsin treatment followed by repeated washes. Thus, only *S. aureus* that has been phagocytosed by the cells is visualized. As shown in Figures 4A, B, senescence dramatically reduced the number of bacteria that were internalized. The first step in the phagocytic process is the particle’s adhesion to the cell membrane. Then, we determine if the decrease in intracellular *S. aureus* is attributable to a failure of bacterial adhesion to the cell. To achieve this, *S. aureus* was incubated at 4°C for 30 min, a temperature that enables binding but prevents internalization. The cells were subsequently fixed, and confocal microscopy was used to evaluate the samples. In senescent cells, we observed significant decreases in the amount of bacteria per cell (Figures 4C, D). These findings demonstrated that inefficient adhesion is likely the cause of the decreased internalization of *S. aureus* by epithelial cells.

All previous experiments in this study were conducted with a cancer-derived cell line. Thus, to have a more physiological model, we used a primary culture of mesenteric artery vascular smooth muscle cells (VSMC). These cells were treated with tBHP as previously described. Subsequently, the cells were infected with *S. aureus* for 2 h, washed, and lysed, and the recovered bacteria from the lysate were quantified using CFUs relative to cell number. Figure 4E demonstrates that, comparable to HeLa cells, in senescent VSMC, a diminished recovery of *S. aureus* was also found.

Professional phagocytes are responsible for eliminating microorganisms within an organism. To determine if senescence has a similar effect on immune cells, we replicated the above assays with Raw 264.7 cells, a monocyte cell line, using the same protocol as with HeLa and VSMC. We determined that senescence also impacts the internalization of bacteria by Raw cells (Figure 4F). Lastly, we wondered whether this process occurs in an organism that gets old. To answer this question, we compared the phagocytosis of macrophages derived from the bone marrow (BMDMs) of 2- and 6-month-old animals. When we infected these BMDMs for 2 h and then counted the recovered bacteria by CFUs after cell lysis, we observed a result that was very similar to that observed with the stress-induced premature senescence model (Figure 4G).

In summary, all these results performed in different cell lines (professional or non-professional phagocytes) support the hypothesis that senescence affects bacterial adhesion to the cells, altering the amount of internalized bacteria. Besides, results primary obtained with culture cells derived from aged organisms show that the phagocytic dysfunction is also observed in aging.

## 4 Discussion

Several studies demonstrate that senescent cells are detectable in tissues, indicating that they persist and accumulate with age (Dimri et al., 1995; Biran et al., 2017; Evangelou et al., 2017)⁠. This indicates that senescent cells may still retain some cellular capabilities. In the current study, we focused on endo/phagocytic activity. First, we observed that senescent cells exhibit an increase in endosomes at each stage of their maturation process. The change in the number of vesicles per cell was partially attributable to an increase in cell size. Nevertheless, neither the maturation nor the degradation capabilities of vesicles were affected. Our findings support the conclusion of Gurley and Dice that there are no differences in the intralysosomal degradation rates of several endocytosed proteins between young and senescent fibroblast cells (Gurley and Dice, 1988)⁠. On the other hand, there are reports that senescent cells undergo receptor-mediated endocytosis changes (Park et al., 2001b; Park et al., 2002)⁠. Noteworthy, these works studied how alterations in the endocytic pathway induce senescence but did not examine how senescence can affect the endocytic pathway (Park, 2002; Shin et al., 2021)⁠. In addition, some studies indicate that senescence influences bacterial phagocytosis but not other intracellular processes, such as superoxide generation (Butcher et al., 2001)⁠. Taken together, the existing information suggests that senescence modifies the internalization of particular cargoes.

According to our study, the recovery of gram-positive and gram-negative bacteria following infection of senescent cells is lower than in normal cells. Interestingly, senescence had little impact on intracellular *S. aureus* trafficking (i.e., colocalization with endocytic markers) or their interactions with autophagy. Interestingly, we demonstrated that senescence affects the internalization stage, particularly bacterial adhesion, in both professional and non-professional phagocytes. Our findings may contribute to explaining why the clinical manifestations of *S. aureus* infection vary between young and elderly people. This notion was strengthened by the results obtained in the BMDM from aged animals, where a defect in the internalization of *S. aureus* was observed in the absence of tBHP treatment.

In young patients, infectious metastases are the most frequent consequences. The proposed process involves bacterial internalization by cells with subsequent intracellular organism dissemination (Mosser and Edwards, 2008)⁠. Although the bacteria are capable of surviving and replicating within cells, this procedure would prevent them from replicating in the bloodstream, where they could cause more severe clinical conditions. A defect in internalization cell capacity may result in an increase in extracellular bacterial replication, thereby promoting the occurrence of a more severe infection. In the present study, we established that senescence influences the internalization of *S. aureus*. As mentioned before, many studies have shown the accumulation of senescent cells in older individuals. This accumulation, in conjunction with the data presented in this report, may explain the high prevalence of *S. aureus* bacteremias and septic shock among the elderly (Gavazzi et al., 2002; Vardi et al., 2013)⁠.

This leads to the idea that senescent cells may play a significant role in aging and disorders associated with aging. Their removal by the immune system may be essential to preserving health (Lujambio, 2016; Kale et al., 2020)⁠. The failure to eliminate senescent cells causes the organism to malfunction (Campisi and D’Adda Di Fagagna, 2007; Kale et al., 2020)⁠. In addition, our findings open the door to novel therapeutic options, such as the use of senolytics to prevent senescent cell accumulation or medications that can affect the surface pattern of senescent cells in order to reverse internalization defects. For this objective, an in-depth understanding of membrane modifications that limit bacterial adhesion is required. Currently, we are focusing on the investigation of several proteins that function as bacterial receptors, such as integrins and multiple TLRs. Also, we are investigating lipid rafts, which have been linked to alterations in receptor-mediated endocytosis (Park et al., 2001a; Park et al., 2001b; Park et al., 2002)⁠. We believe that the findings of our current and future investigations will increase the general understanding of infectious diseases.

## Data Availability

The original contributions presented in the study are included in the article/Supplementary Material, further inquiries can be directed to the corresponding authors.

## Supplementary material

SUPPLEMENTARY FIGURE S1 Senescence has no effect on endosome maturation. **(A)** HeLa cells treated or untreated with t-BHP, fixed and immunostained with an anti-LAMP2 antibody and a specific secondary antibody. (Scale bar, 10 μm). **(B)** The number of LAMP2 positive dots was quantified [*n* = 3 (each n tested = 30 cells), mean SEM ****p* 0.0001]. **(C)** Quantification of LAMP2 positive vesicles in control and senescent cells per cell relative to the cell size (Mean ± SEM ns *p* = 0.8752). **(D)** Quantification of GFP-Rab 7 positive vesicles in control and senescent cells per cell relative to the cell size (Mean ± SEM ns *p* = 0.1073). **(E)** Both t-BHP-treated and untreated cells were transfected with GFP-Rab7, and 24 h after transfection, cells were fixed and immunostained with an anti-LAMP2 antibody and a specific secondary antibody. (Scale bar, 10 μm). **(F)** Samples were analyzed by confocal microscopy, and colocalization of GFP-Rab7 with LAMP2 was calculated [*n* = 3 (each n tested = 40 cells), mean ± SEM ns *p* = 0.2075].

SUPPLEMENTARY FIGURE S2 Senescence does not alter cathepsinD processing. **(A)** Process of conversion of pro-cathepsin D to cathepsin D in endo/lysosomal compartments. **(B)** HeLa cells treated or untreated with t-BHP were processed for Western blot with an antibody against cathepsinD. **(C)** Quantification of cathepsinD intensity over pro-cathepsinD intensity (*n* = 3, mean ± SEM ns *p* = 0.2558).

SUPPLEMENTARY FIGURE S3 Senescence modify *S. aureus* internalization but not intracellular trafficking. **(A)** Confocal microscopy of control and senescent cells, transfected with Rab5, infected with *S. aureus*, and fixed at 1 h.p.i. M.O.I. = 10 (scale bar= 10 um). **(B)** Quantification of the proportion of bacteria present on GFP-Rab5 positive vesicles in control and senescent cells. (Mean ± SEM ns *p* = 0.9881). **(C)** Cells treated with t-BHP or untreated were pre-incubated for 1 h with Bafilmycin A1 and infected with *S. aureus* M.O.I. = 10. Subsequently, they were processed by Western blot to detect LC3 or actin. One image representative from three experiments.
