# Supplementary figures and images for: Staphylococcus aureus phagocytosis is affected by senescence
Source: Front Aging. 2023 Jul 31;4:1198241. doi: 10.3389/fragi.2023.1198241 (PMC10423838; doi:10.3389/fragi.2023.1198241)

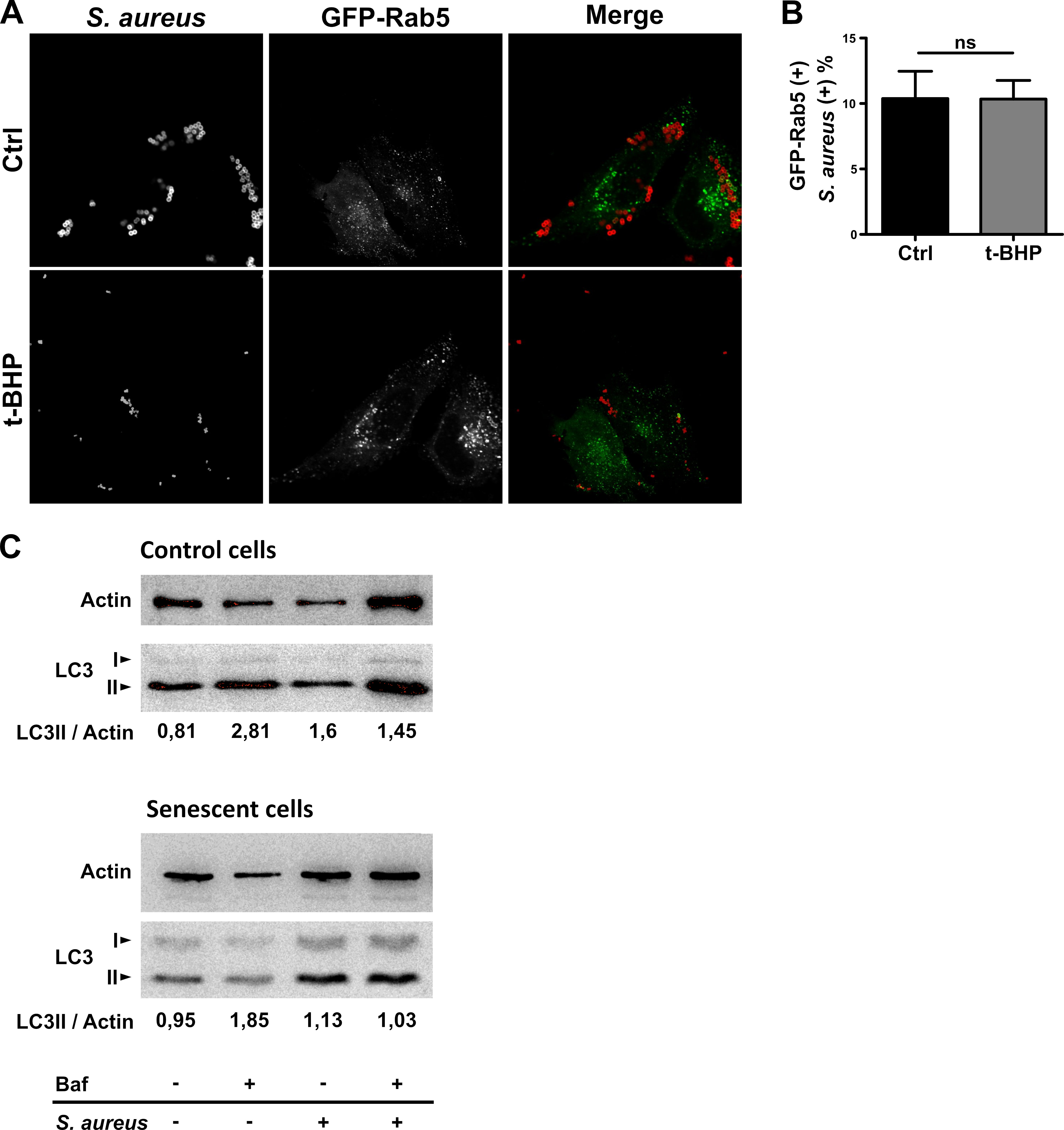

Supplement: Supplementary file 1 [file Image3.JPEG]

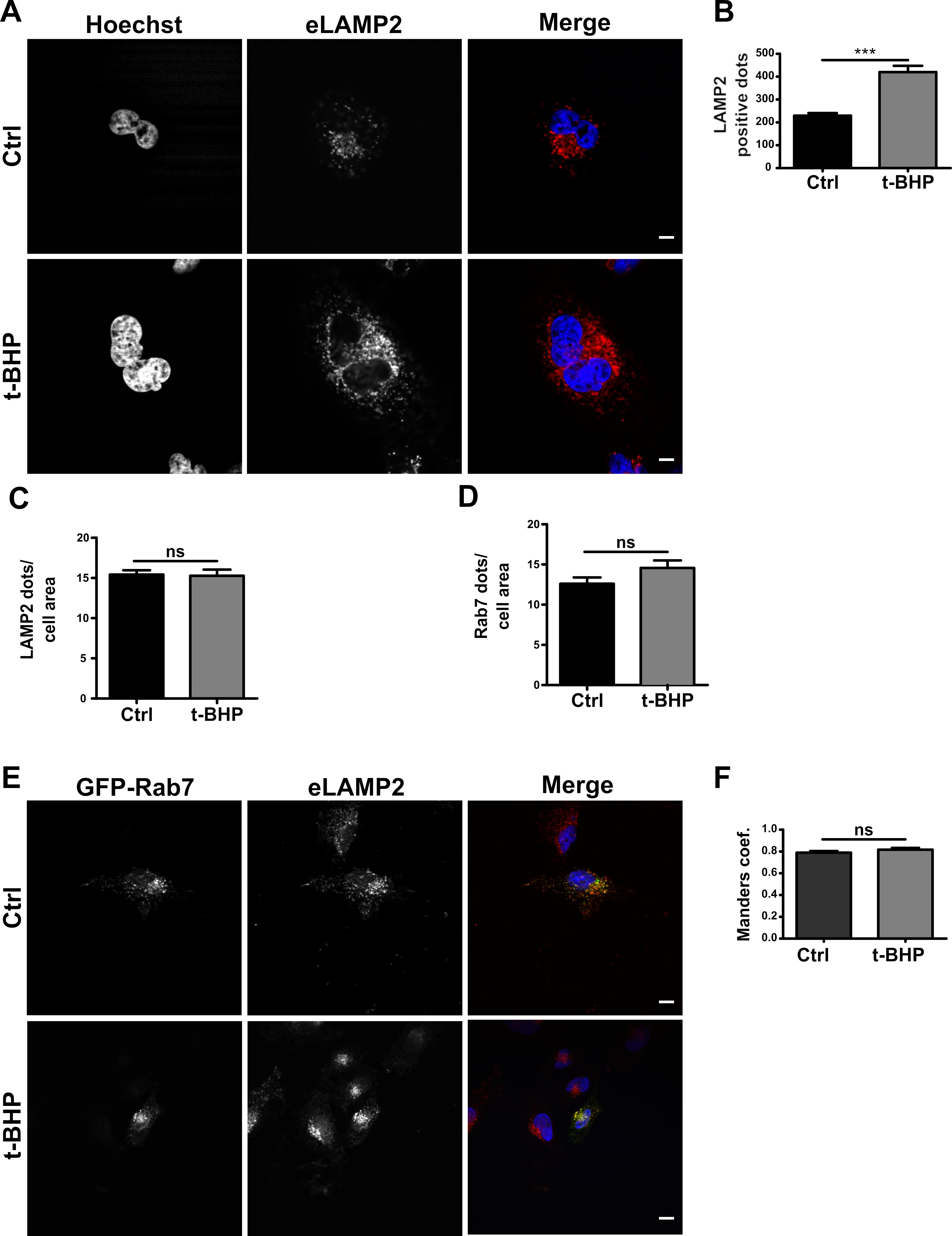

Supplement: Supplementary file 2 [file Image1.JPEG]

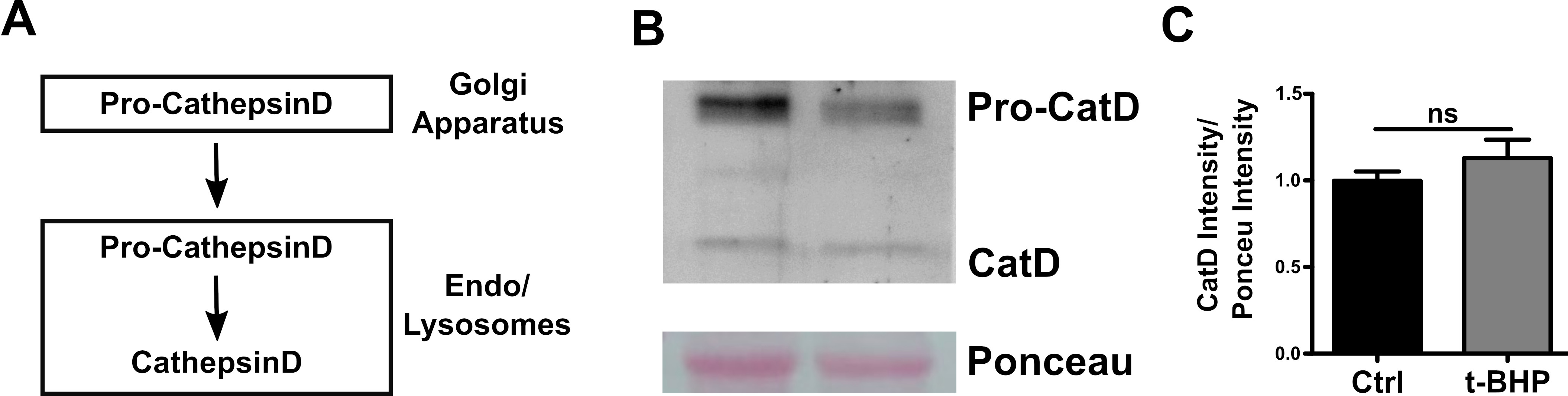

Supplement: Supplementary file 3 [file Image2.JPEG]
